# Community Health Worker–Integrated Model for Sickle Cell Disease Management: Protocol for a Feasibility Study

**DOI:** 10.2196/82663

**Published:** 2026-03-26

**Authors:** Tilicia Mayo-Gamble, Doreen Ugwu, Tobi Oloyede, Joseph Telfair, Abdullah Kutlar

**Affiliations:** 1 Health Policy and Community Health, Jiann-Ping Hsu College of Public Health, Georgia Southern University, 501 Forest Dr, Statesboro, GA, 30458, United States, 1 912-478-1249; 2 Center for Blood Disorders, Medical College of Georgia, Augusta University, Augusta, GA, United States

**Keywords:** sickle cell disease, community health worker, patient-centered care, self-care, mixed methods, research protocol

## Abstract

**Background:**

Sickle cell disease (SCD) is a chronic inherited blood disorder that disproportionately affects African Americans. Managing SCD is complex, often requiring coordination of care among multiple health care professionals. These care challenges result in fragmented service delivery and poor health outcomes. Integrating community health workers (CHWs) into patient-centered coordinated care may enhance disease management and patient satisfaction with the care received.

**Objective:**

The proposed study aims to test the feasibility and acceptability of a CHW-integrated model for SCD management.

**Methods:**

This protocol is for a multimethod study to evaluate the feasibility of an integrated CHW intervention. The intervention includes CHW-led patient education and care coordination. Adults with SCD (aged 18‐45 years) will be recruited from the Augusta University Sickle Cell Center’s satellite clinic in Albany, Georgia. Quantitative outcomes include self-care behaviors and patient satisfaction. Qualitative data from semistructured interviews will be used to assess the acceptability of the intervention.

**Results:**

The proposed study will provide preliminary data on the impact of a CHW-integrated model on care coordination and self-management among adults with SCD. As of March 2026, we have enrolled 16 participants.

**Conclusions:**

Despite national calls for improved care coordination and disease management in this population, few published protocols detail community-based, culturally aligned approaches that center on the lived experiences and structural barriers faced by adults with SCD. Our study addresses a gap in the literature by highlighting a theory-informed, multimethod study protocol for evaluating the feasibility, effectiveness, and acceptability of integrating CHWs into a patient-centered coordinated care model for adults with SCD. This protocol outlines a scalable, community-embedded strategy that may inform future clinical trials and implementation efforts.

## Introduction

Sickle cell disease (SCD) is an inherited hemoglobin disorder that affects 1 in 365 African Americans [1,2]. Disparities in receiving health care among African Americans and other racial/ethnic minority groups in the United States are well documented and are directly related to poor health outcomes associated with SCD. Individuals with SCD experience physical, psychological, and social comorbidities that impact effective disease management [3,4]. The challenges of providing care to this population with complex needs have led to fragmented care, poor patient experiences, and inadequate implementation of evidence-based SCD management [5,6]. In 2014, the National Heart, Lung, and Blood Institute released a report on “Evidence-Based Management of Sickle Cell Disease” [7], calling for strategies to improve disease management by patients and providers [8]. Patient-centered coordinated care (PCCC) is an evidence-based strategy that can potentially reduce care fragmentation and improve patient experiences. However, studies report deficiencies in care coordination and patient satisfaction among individuals with SCD [9,10]. These deficiencies contribute to the inability of African Americans with SCD to obtain consistent, preventive care, resulting in poorer physical and psychological health outcomes.

Georgia ranks among the top 3 states in the prevalence of SCD, affecting more than 10,000 individuals, including a significant adult population, across nearly every county [11]. Access to specialized care constitutes a significant barrier for these individuals, as data from the Sickle Cell Data Collection program reveal that 10% of babies born with SCD between 2004 and 2016 lived more than 1 hour away from the nearest specialty care center, a situation that contributes to delays in treatment and increased health care use that can persist into adulthood [11]. Many adults with SCD experience challenges related to treatment adherence and health care access, which underscores the urgent need for community-based interventions aimed at enhancing support for this population. To address these barriers, the Georgia Senate Study Committee on Sickle Cell Disease recommended expanding community-based solutions, such as leveraging Federally Qualified Health Centers, mobile health units, and telemedicine—approaches that align with the integration of community health workers (CHWs) to support disease management [12].

A community-based approach could bridge gaps in health care services to improve patient care for SCD. CHWs have demonstrated promise in improving health behaviors and outcomes among racial and ethnic minority populations with chronic diseases who lack access to adequate health care [13,14]. CHWs can be integrated into a PCCC team to enhance care coordination and facilitate disease management among adults with SCD. This evidence-based strategy is supported by the Sickle Cell Disease Association of America, a national advocacy organization for the SCD community [15]. Studies have demonstrated that both PCCC and CHW interventions are effective models for supporting disease management in chronic conditions [16,17]. However, there are few models for integrating CHWs into a PCCC team to enhance disease management and patient experiences [17,18]. Furthermore, no published studies have used such an integrative model among African American adults with SCD.

This research will build on our understanding of the role of CHWs in the evidence-based management of SCD and will identify challenges associated with integrating a CHW into a clinical care team. To realize the tenets of this hypothesis, three specific aims will be pursued: (1) assessment of the feasibility of integrating a CHW into a PCCC team for SCD, (2) evaluation of the effectiveness of enhanced care coordination using an integrated PCCC-CHW model on self-care behaviors and patient satisfaction, and (3) determination of the acceptability of the intervention and protocol among patients and care team members through semistructured interviews. Our study will evaluate the hypotheses that by integrating a CHW into a PCCC team, barriers to disease management can be overcome while simultaneously improving the quality of care for adults with SCD.

## Methods

### Overview

The chronic care model (CCM) is a comprehensive framework designed to shift the management of chronic diseases from a reactive approach to a more proactive, planned strategy that emphasizes continuous patient-provider relationships, individualized care, and evidence-based practices across health care systems [19]. It identifies elements of a health care system that encourage high-quality chronic disease care. The CCM posits that productive interactions should occur between patients and providers [19]. However, the framework is limited in describing processes for facilitating these interactions. The proposed study will examine the effectiveness of integrating a CHW as a liaison between patients and providers. This integration is expected to enhance care coordination and support chronic disease management, particularly among adults affected by SCD [13,14].

The theory of self-care management for SCD is adapted from the broader theory of self-care management for vulnerable populations, which describes variables that influence self-care management, health status, and quality of life among populations that experience or are at risk for health disparities [20]. This theory underscores the significance of self-care practices in managing chronic disease, particularly by helping individuals navigate the demands of their condition. In this research, the self-care construct from the theory of self-care management for SCD will be applied as the self-management support element of the CCM, thereby fostering self-care behaviors to improve health outcomes among individuals with SCD.

**Figure 1.**
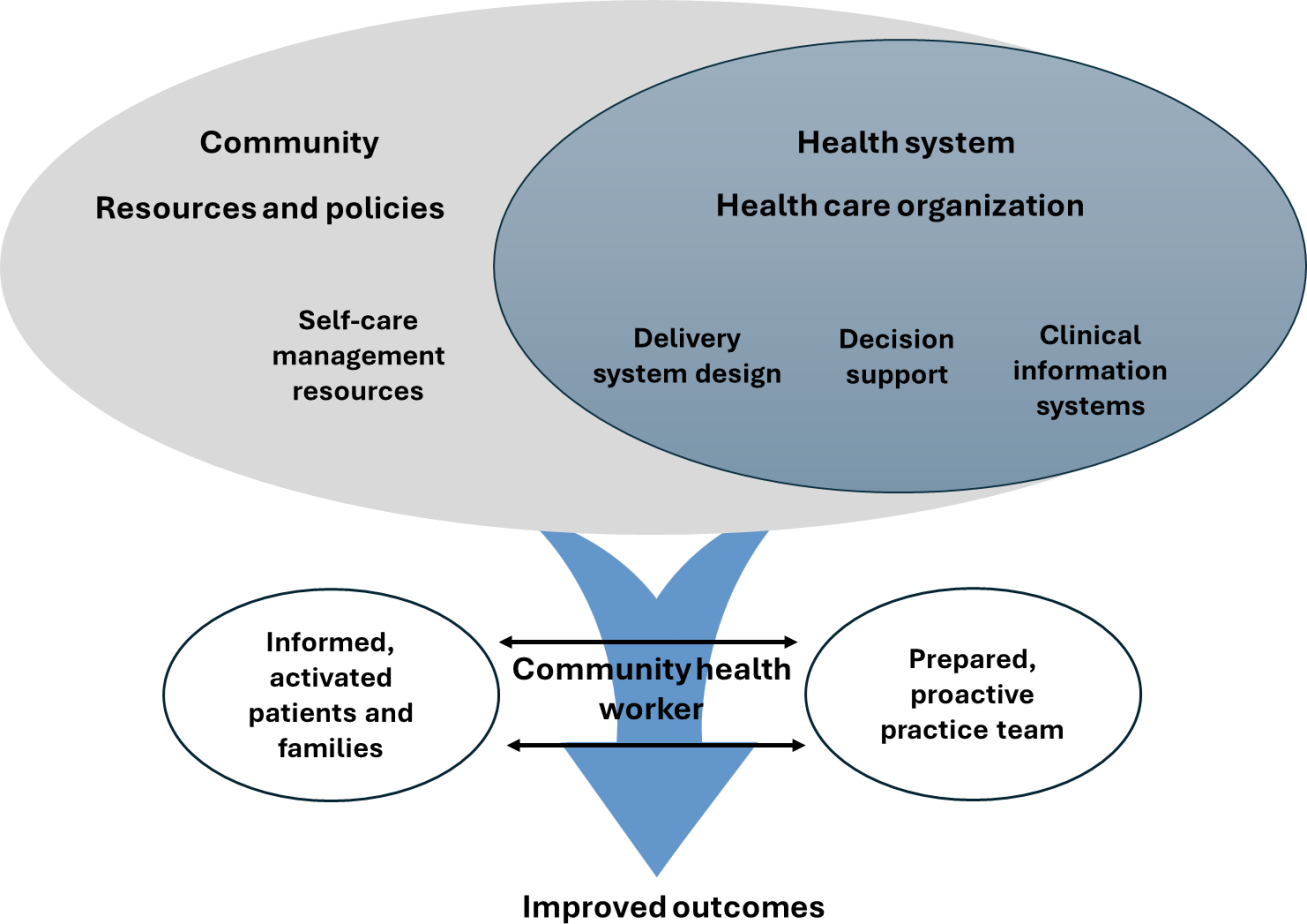
Adapted version of the chronic care model.

The goal of this research protocol is to evaluate the feasibility and effectiveness of integrating a CHW into a PCCC team to enhance self-care for adults with SCD. Adopting the CCM and the theory of self-care management for SCD, we hypothesize that a CHW can be successfully integrated into a PCCC team to overcome barriers to disease management and improve the quality of care for adults with SCD (Figure 1).

Figure 1 illustrates the proposed integrated model. The CCM identifies elements of a health care system that encourage high-quality chronic disease care and emphasizes productive interactions between informed, activated patients and prepared, proactive care teams [19]. For the proposed model, we will assess the effectiveness of a CHW as a liaison to facilitate productive interactions between patients and providers [16-18]. To strengthen theoretical alignment with SCD-specific care needs, the self-management support component of the CCM is operationalized using the theory of self-care management for SCD, which specifies individual-level processes that influence self-care behaviors and health outcomes in this population [20]. Within this integrated framework, CHWs will support self-care skill development, reinforce patient engagement, and facilitate coordination among patients, providers, and community resources. The proposed study will use a CHW to offer social support and implement evidence-based guidelines through virtual or telephone-based sessions. This approach will allow patients to remain in their home setting, where they experience the daily challenges of self-management.

### Ethical Considerations

This protocol will be conducted in accordance with the Declaration of Helsinki. Ethics approval has been granted on an expedited basis by the institutional review board of Georgia Southern University (H21406) for surveys and interviews conducted with human subjects. Informed consent will be obtained from all participants, who will then receive a link to the baseline questionnaire to be completed via Qualtrics XM (Silver Lake and CPP Investments). Each participant will receive a US $20 gift card after completing the questionnaire.

Health Insurance Portability and Accountability Act (HIPAA)–protected information will not be discussed during calls. Information collected during calls will be stored on a cloud server that is only accessible to the research team. To protect the confidentiality of participants, each of them will be assigned a unique participant code.

### CHW Training

In a prior study, the lead investigator conducted a patient-centered outcomes project that trained CHWs on evidence-based management for SCD and the facilitation of education on SCD management for patients and caregivers [21]. On the basis of the study’s findings, we anticipate that participants will perceive CHWs as an acceptable method for delivering SCD education. CHWs (n=2) will be recruited from My Three Sicklers Sickle Cell Foundation Inc (MTS), a Georgia-based community organization that focuses on SCD outreach, management, and policy.

For training, CHWs will complete a “Remote Community Health Worker Certificate” via HomeTown Health, a network of rural hospitals that provides continuing education and training for health care providers. This training provides CHWs with foundational knowledge on the role of CHWs and the delivery of education in remote settings. Once completed, the principal investigator (PI) will provide project onboarding and training for SCD management. MTS will provide ongoing support and supervision of CHWs throughout the study duration.

### Study Design, Setting, and Sample

**Figure 2.**
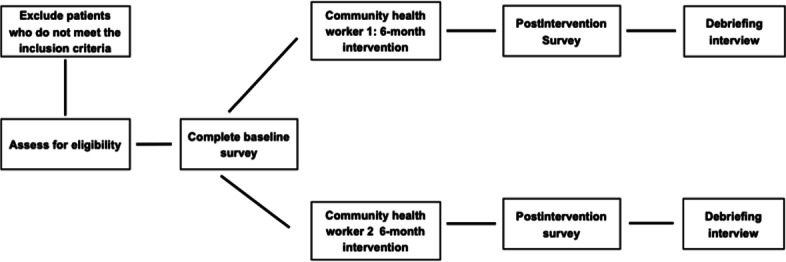
Sickle cell disease community health worker intervention flowchart.

This study will use a pre-post, nonrandomized, 6-month longitudinal intervention design, implemented using questionnaires and semistructured interviews (Figure 2). Enrollment will occur through the Augusta University Sickle Cell Center’s satellite clinic, Phoebe Putney Cancer Center, in Albany, Georgia. This site has not previously worked with a CHW, either directly or through a community-based organization.

Eligible participants will be adults with SCD aged 18‐45 years. Given the target sample size (n=40), at 80% power and α=.05, we can detect small to moderate effect sizes (*r*^2^=0.4 to 0.6) using a 2-tailed paired *t* test [22]. This sample includes an expected 20% attrition rate. There are currently 97 eligible individuals seeking SCD care at this location. This is more than double the expected enrollment of 30 participants. Using a rolling recruitment process, the PI will work with MTS to enroll additional participants if an enrolled participant is lost to follow-up. The assumption of a small to moderate effect is conservative, given that studies designed to improve self-care in SCD report a medium effect size (*d*=0.71) [8,23].

### Procedures and Data Collection

The primary outcomes for this study are patient satisfaction and self-care behaviors (including appointment keeping, tracking health issues, and managing medications). These will be assessed using validated instruments at baseline and after intervention (following the 6-month CHW-integrated PCCC model). Feasibility will be assessed through (1) enrollment numbers within a 2-year time frame, (2) retention, (3) intervention completion, and (4) acceptability of the intervention among participants.

### Study Procedures

Adults with SCD (aged 18‐45 years) will be recruited from the Augusta University Sickle Cell Center’s satellite clinic in Albany, Georgia. Clinic staff will identify participants who meet the inclusion criteria. For eligible patients, clinic staff will describe the study and offer participation during the patient’s regularly scheduled clinic visit. During the visit, interested patients will provide written informed consent and the link to the baseline questionnaire to be completed via Qualtrics. Each participant will receive a $20 gift card after completing the questionnaires. Following enrollment, participants will be referred to MTS for assignment to a CHW.

The CHW will schedule an initial session (60 minutes) with participants, followed by 5 subsequent monthly sessions (virtual or telephone-based) scheduled for 30 to 60 minutes, for a maximum of 6 patient contact hours over 6 consecutive months. During each monthly session, the CHW will educate participants on SCD management (ie, preventive services, management of acute complications, and management of chronic complications). The CHW will use a subjective, objective, assessment, and plan (SOAP) format for reporting via Qualtrics. The SOAP form is a standardized documentation method used by health care providers to systematically record patient information, thereby enhancing communication about health care experiences [24]. By organizing patient encounters into 4 distinct sections, the SOAP note serves as a clear framework for evaluating patient data and outlining management strategies [24]. This report will allow the CHW to document observations from patient interactions.

After completion of the sixth and final session, the CHW will provide a link to the postintervention questionnaire to be completed via Qualtrics. The postintervention questionnaire contains a question of interest about participating in debriefing interviews. We will conduct semistructured debriefing interviews with patients (n=35 approximately) to determine the perceived feasibility and acceptability of the study procedures. A member of the research team will conduct the interviews using a semistructured interview guide. Interviews are expected to last 45 to 60 minutes, and compensation will be a US $25 gift card. All incentives will be distributed by the PI per the institutional review board–approved protocol for research involving human subjects.

### Measures

#### Overview

Eligible patients will be referred to MTS for assignment to a CHW. At the initial session, participants will complete a baseline survey assessing patient satisfaction and self-report self-care behaviors. We will use a demographics questionnaire, the Medical Outcomes Study Patient Satisfaction Questionnaire [25], and the Jenerette Self-Care Assessment Tool [20] (refer to Multimedia Appendix 1 for details). The CHW will complete a SOAP report at each session, documenting observations of the participant’s condition and responses related to self-care behaviors. After each visit, the CHW will report the results to the PI. These sessions will allow the PI to assess study fidelity and address any challenges with implementation or potential participant dropout. It will also allow the research team to determine whether modifications to the program are needed to ensure participant retention. During the final CHW session, the participant will complete a postintervention survey on patient satisfaction and self-report self-care behaviors.

Patient satisfaction with medical care will be assessed using the Medical Outcomes Study’s Patient Satisfaction Questionnaire Short Form (PSQ-18) [25]. The PSQ-18 is an 18-item self-report instrument designed to evaluate patients’ satisfaction with health care services across multiple domains, including general satisfaction, technical quality, interpersonal manner, communication, financial aspects, time spent with providers, and accessibility and convenience. Items are rated on a 5-point Likert scale ranging from strongly disagree to strongly agree. Subscale scores are computed by averaging item responses, with higher scores indicating greater satisfaction with care. The PSQ-18 has demonstrated good reliability and validity across diverse patient populations and is widely used in health services research [25].

SCD-specific self-care behaviors will be assessed using the Jenerette Self-Care Assessment Tool [20]. This instrument was developed to evaluate self-care management behaviors among individuals with SCD, including symptom monitoring, pain management, medication adherence, and engagement in health-promoting behaviors. The tool consists of multiple self-report items rated on a Likert-type scale, with higher scores indicating greater engagement in effective self-care behaviors. The Jenerette Self-Care Assessment Tool has demonstrated acceptable reliability and validity and has been used in previous studies examining self-care management among adults with SCD [20].

#### Acceptability of the Intervention

To determine the acceptability of the protocol, we will use semistructured interviews to determine patient satisfaction with the intervention protocol and specific components (clarity, ease of use, relevance, helpfulness, and new information), actual time required, trust or rapport with the CHW, influence on self-care, and suggestions for improvement. Additionally, research staff will conduct interviews with the CHWs (n=2) and clinic staff (n=3). Interviews are expected to last 30 minutes.

### Data Analysis

SPSS (version 25; IBM Corp) will be used to analyze quantitative data collected from the survey instruments. Categorical data will be described using frequencies and percentages. Continuous measures will be described using means and SDs. Cronbach α will be used to calculate the internal consistency for the instruments [26]. To examine relationships between measures, *χ*^2^ tests will be used for categorical variables, paired *t* tests will be used to compare pre-post measures, and ordinary least squares multiple regression analyses will be conducted to examine associations between demographic variables and outcome measures. Missing data will be minimized using stop measures including using Qualtrics to prevent skipping questions and applying case deletion for surveys with missing entries that are important to answer the primary research question.

NVivo (version 15; Lumivero) will be used to analyze qualitative data collected through semistructured interviews. Data will be transcribed, deidentified, and coded for analysis [27]. To ensure rigor, we will work with another member of the research team associated with the parent grant to independently analyze the transcripts. Findings from the 2 independent analyses will be reviewed and refined until agreement is reached [28]. Findings will be reviewed for accuracy of data interpretation, themes generated, and conclusions drawn.

## Results

Quantitative and qualitative results will be triangulated. The qualitative debriefing interviews will allow us to expand on implementation and practical challenges. We will also identify areas for improvement regarding the intervention logistics and patient recommendations in preparation for a larger study. A challenge of this novel approach is the limited precedent for integrating a CHW to supplement SCD specialty care. However, this study is expected to yield important preliminary data for developing an optimally effective intervention to be tested in a future feasibility study. As of March 2026, we have enrolled 16 participants.

## Discussion

This research protocol outlines a novel approach to enhancing care coordination and self-care management for adults with SCD through the integration of CHWs into a PCCC team. The methodology draws inspiration from established models for chronic disease management and adapts them to the specific needs of adults with SCD in Georgia, a state with a high prevalence of SCD [11].

The proposed intervention leverages the known effectiveness of CHW support in managing chronic conditions, particularly among racial and ethnic minority groups who experience health care disparities [13]. Similar to studies with a focus on improving adherence to hydroxyurea among adolescents with SCD [29,30], our protocol recognizes the value of community-based approaches to address barriers to care. The use of a CHW as a liaison between patients and providers is a key element of this approach, aiming to bridge gaps in fragmented care and foster productive interactions, as highlighted by the CCM [19].

While previous studies have demonstrated the effectiveness of CHWs in various chronic disease contexts, including preliminary work in SCD [16,29], there remains a need for empirical evaluation of integrated PCCC-CHW models specifically for African American adults with SCD. The inclusion of semistructured interviews will offer qualitative insights into the acceptability of the intervention from the perspectives of patients and the care team, which is a vital step toward developing sustainable and patient-centered programs. Additionally, this study supports the standardization of CHW training and certification for SCD management.

This study acknowledges potential limitations, including the feasibility of the sample size, which, while appropriate for evaluating the protocol’s implementation, may limit statistical power to detect significant differences in outcomes. Larger studies using similar protocols would provide concrete data to demonstrate that CHWs are essential contributors to interdisciplinary teams that care for patients with a broad spectrum of chronic diseases. However, the multimethod approach to data collection, incorporating both quantitative measures and qualitative feedback, is a strength that will provide a comprehensive understanding of the intervention’s impact and inform necessary refinements for future research. The focus on adults is particularly relevant given the unique challenges this population faces in accessing consistent and specialized care, especially in areas with geographic disparities [31].

## Supplementary material

10.2196/82663 Multimedia Appendix 1 Integrated intervention measures.

## Abbreviations

CCM: chronic care model
CHW: community health worker
HIPAA: Health Insurance Portability and Accountability Act
MTS: My Three Sicklers Sickle Cell Foundation Inc
PCCC: patient-centered coordinated care
PI: principal investigator
PSQ-18: Patient Satisfaction Questionnaire Short Form
SCD: sickle cell disease
SOAP: subjective, objective, assessment, and plan
