# Supplementary material for: Community Health Worker–Integrated Model for Sickle Cell Disease Management: Protocol for a Feasibility Study
Source: JMIR Res Protoc. 2026 Mar 26;15:e82663. doi: 10.2196/82663 (PMC13021104; doi:10.2196/82663)
Supplement: Multimedia Appendix 1 [file resprot-v15-e82663-s001.docx]

| Supplemental Table: Integrated intervention measures | | | | |
| --- | --- | --- | --- | --- |
| **Primary Outcomes** | **Assessment** | **No. of Questions** | **Assessment Method** | **Example of Item** |
| 1. Self-care Action | JSAT/SOAP  (The following questions are about your self-care related to managing your sickle cell disease.) | 8 | 4-point frequency scale (Never, Rarely, Sometimes, Almost Always) | "I take medications as prescribed." |
|  |  |  |  | “I drink plenty of fluids.” |
| 2. Patient Activation | The following questions are about your disease management activities. | 13 | 5-point Likert scale (Strongly Disagree, Disagree, Neutral, Agree, Strongly Agree) | “I am confident that I can tell when I need to go get medical  care and when I can handle a health problem myself.” |
|  |  |  |  | “I know what each of my prescribed medications do.” |
| 3. Self-Efficacy | The following questions are about your self-care related to managing your sickle cell disease. | 9 | 5-point scale (Not at All Sure, Not Sure, Neither, Sure, Very Sure) | “How sure are you that you can control how often or when you get tired?” |
| 4. Patient Satisfaction | PSQ  (The following questions are about your satisfaction with the medical care you receive for your  sickle cell disease.) | 10 | 5-point Likert scale (Strongly Disagree, Disagree, Neutral, Agree, Strongly Agree) | “I am very satisfied with the medical care I receive.” |
|  |  |  |  | “I am able to get medical care whenever I need it.” |
